# Gesundheitsökonomische Evaluation einer rehabilitativen Kurzzeitpflege: Ermittlung der Kosten von geriatrischen Patienten mit und ohne rehabilitative Kurzzeitpflege nach stationärem Krankenhausaufenthalt

**DOI:** 10.1007/s00391-024-02307-2

**Published:** 2024-05-15

**Authors:** S. Diekmann, P. zur Nieden, K. Pahmeier, J. Frankenhauser-Mannuß, A. Keilhauer, N. Specht-Leible, J. Bauer, T. Hüer, P. Raszke, A. Walendzik, J. Wasem, A. Neumann

**Affiliations:** 1 Essener Forschungsinstitut für Medizinmanagement (EsFoMed) GmbH, Essen, Deutschland; 2 Unternehmensbereich Versorgungsgestaltung, Geschäftsbereich Versorgungsinnovationen & sektorenübergreifende Versorgungslösungen, AOK Baden-Württemberg, Stuttgart, Deutschland; 3 Geriatrisches Zentrum, Universitätsklinikum Heidelberg, AGAPLESION Bethanien Krankenhaus Heidelberg, Heidelberg, Deutschland; 4 Lehrstuhl für Medizinmanagement, Universität Duisburg-Essen, Thea-Leymann-Str. 9, 45127 Essen, Deutschland

**Keywords:** Rehabilitation, Poststationäre Versorgung, Kostenanalyse, Abrechnungsdaten, Geriatrie, Rehabilitation, Post-inpatient care, Analysis of costs, Health administrative data, Geriatrics

## Abstract

**Hintergrund:**

Geriatrische Patient:innen mit Rehabilitationsbedarf, die im Anschluss an einen akutstationären Aufenthalt in Kurzzeitpflege (KZP) gehen, erhalten selten Rehabilitation. Die rehabilitative Kurzzeitpflege (REKUP) erweitert die KZP um rehabilitative Maßnahmen, u. a. um Dauerpflege (DP) zu vermeiden.

**Ziel der Arbeit:**

Eine Kosten- und Kosten-Effektivität-Analyse sollen Informationen für eine flächendeckende Anwendung liefern.

**Material und Methoden:**

Mittels einer nichtrandomisierten, kontrollierten prospektiven Studie wurde REKUP erprobt. Der Interventionsgruppe (IG) wurde eine Kontrollgruppe (KG) mittels 1:2-Matching zugewiesen, wobei 3 Kollektive (jeweils IG und KG) gebildet wurden, mit Nachbeobachtungszeiträumen von 30, 90 und 180 Tagen. Die durchschnittlichen Gesamtkosten aus Kostenträgerperspektive wurden anhand von Abrechnungsdaten der AOK Baden-Württemberg ermittelt. Ein möglicher Einfluss der Intervention auf die Kosten wurde unter Verwendung des Difference-in-difference-Ansatzes analysiert.

**Ergebnisse:**

Die Analyse schließt 43 (IG) und 86 (KG) geriatrische Patient:innen ein. Im Postzeitraum nahmen Patient:innen der IG häufiger eine Reha in Anspruch und gingen weniger häufig in DP bzw. verstarben. Die Analyse der Kosten im Postzeitraum zeigte in allen Kollektiven keinen statistisch signifikanten Unterschied zwischen IG und KG. Für Pflege und Arzneimittel waren im Postzeitraum die Kosten der KG, im Bereich der Rehabilitation die Kosten der IG statistisch signifikant höher (*p* < 0,001).

**Diskussion:**

Patient:innen der IG hatten bei gleichen Kosten Vorteile in Bezug auf die Inanspruchnahme von Rehabilitation, Vermeidung von DP und Versterben. Dies weist auf eine mögliche Vorteilhaftigkeit von REKUP in der Zielpopulation hin, die aufgrund methodischer Einschränkungen weiter erforscht werden sollte.

**Zusatzmaterial online:**

Zusätzliche Informationen sind in der Online-Version dieses Artikels (10.1007/s00391-024-02307-2) enthalten.

## Abstract (en)

**Background:**

Geriatric patients requiring rehabilitation and admitted to short-term care after an acute inpatient hospital stay seldom receive rehabilitative services later. Rehabilitative short-term care (REKUP) supplements short-term care with rehabilitative measures, aiming to prevent functional restrictions and long-term care.

**Study objective:**

To conduct a cost and cost-effectiveness analyses of REKUP and provide data for a nationwide rollout.

**Material and methods:**

A non-randomized controlled prospective study was carried out. The intervention group (IG) was paired 1:2 with a control group (KG), resulting in the formation of three collectives with follow-up periods of either 30, 90 or 180 days (each with IG and KG). Using administrative claims data from the AOK Baden-Württemberg, the mean total costs from the perspective of the health insurance were calculated. A potential impact of the intervention on costs was analyzed using the difference in differences approach.

**Results:**

The analysis comprised 129 patients (IG 43; KG 86). During the follow-up periods, the IG presented higher rates of rehabilitation and lower rates of long-term care and mortality. Regarding costs, no statistically significant differences were found between the IG and KG in any of the three collectives. For nursing care and medication costs, costs were significantly higher in the follow-up period for the KG, whereas costs for rehabilitation were significantly higher for the IG (*p* < 0.001).

**Discussion:**

Patients receiving REKUP utilize rehabilitation services more often and have a lower likelihood of requiring nursing care or dying with no statistically significant differences in costs. There are potential advantages of REKUP in the target population, which warrant further investigation due to methodological limitations.

Geriatrische Patient:innen in Kurzzeitpflege (KZP) mit Rehabilitationsbedarf nach akutstationärem Aufenthalt nehmen selten eine Rehabilitation in Anspruch, was langfristig zu einem erhöhten Bedarf an Pflegeleistungen führen kann. Die rehabilitative Kurzzeitpflege (REKUP) wurde entwickelt, um die poststationäre Versorgung zu verbessern und eine Überleitung in die Rehabilitation zu fördern. Eine gesundheitsökonomische Evaluation kann bei der Entscheidung zur Implementierung dieses neuen Versorgungsmodells helfen.

## Hintergrund

Die Inanspruchnahme der KZP von geriatrischen Patient:innen nach einem Krankenhaus(KH)-Aufenthalt steigt [2]. In Anbetracht demografischer Entwicklungen und eines zu erwartenden Anstiegs Pflegebedürftiger bis 2030 auf rund 6 Mio. [11] erhalten Aufrechterhaltung der Selbstständigkeit und Mobilität, die möglichst eigenständige Alltagsbewältigung sowie Verhinderung/Verminderung von Pflegebedürftigkeit zunehmend Relevanz [1].

Etwa 44 % der geriatrischen Patient:innen in KZP, die nach einem KH-Aufenthalt entlassen werden, haben einen Rehabilitationsbedarf [6]. Trotz Rehabilitationspotenzial und -bedarf nehmen nur wenige Rehabilitation in Anspruch, da es oft an Konzepten für den Übergang zwischen KH-Aufenthalt und Rehabilitation mangelt [3]. Die Institutionalisierung im Krankenhaus kann eine Rückkehr in die Häuslichkeit erschweren [10].

Die Förderung von Potenzialen nach einem KH-Aufenthalt ist entscheidend für die Rückkehr von geriatrischen Patient:innen in die Häuslichkeit sowie für die Vermeidung/Verringerung von Pflegebedürftigkeit [3, 8]. Bisher gibt es wenige Standards oder gesetzliche Vorgaben für die poststationäre Versorgung von geriatrischen Patient:innen [14]. Die geringe Inanspruchnahme von Rehaleistungen und die niedrigen Rücküberleitungsquoten in die Häuslichkeit verdeutlichen den Bedarf an Verbesserungen in der poststationären Versorgung dieser Zielgruppe [3].

Die REKUP wurde entwickelt, um die poststationäre Versorgung von geriatrischen Patient:innen mit Rehabilitationsbedarf, aber ohne bestehende Rehabilitationsfähigkeit zu verbessern. Das Modell zielt darauf ab, funktionelle Einschränkungen zu stabilisieren, die Überleitung in Rehabilitation zu fördern und Pflegebedürftigkeit zu vermeiden/zu verringern. Somit folgt REKUP dem Prinzip „Rehabilitation vor Pflege“ [15].

## Fragestellung

Aufgrund der zu erwartenden steigenden Zahl Pflegebedürftiger sowie steigenden Ausgaben der Pflegeversicherung [13], gewinnen eine fachspezifische Versorgung von geriatrischen Patient:innen und eine nachhaltige Finanzierung an Bedeutung. Mittels Kosten- und Kosten-Effektivität(KE)-Analyse sollen die monetären Auswirkungen von REKUP im Vergleich zur Regelversorgung aufgezeigt werden.

## Studiendesign

Die Intervention REKUP wurde anhand einer nichtrandomisierten, kontrollierten prospektiven Studie in Baden-Württemberg (BW) erprobt und durch die Ethikkommission der Landesärztekammer BW genehmigt. Die Durchführung erfolgte im Einklang mit nationalem Recht sowie gemäß der Deklaration von Helsinki von 1975 (in der aktuellen, überarbeiteten Fassung). Eine Einverständniserklärung von allen Patient:innen liegt vor. Die gesundheitsökonomische Evaluation (GE) erfolgte aus Kostenträgerperspektive. Die Darstellung der methodischen Vorgehensweise erfolgte in Anlehnung an die Empfehlungen der Consolidated Health Economic Evaluation Reporting Standards [5].

### Zielgruppe

Zielgruppe für die Erprobung waren bei der AOK BW Versicherte, geriatrische Patient:innen (≥ 70 Jahre), die nach akutstationärer Versorgung in KZP entlassen wurden, rehabilitationsbedürftig, aber nicht rehabilitationsfähig waren sowie ein konkretes Rehabilitationsziel hatten. Eine Einschränkung hinsichtlich des Indikationsgebiets erfolgte nicht. Die Zielgruppe wurde gemäß den Kriterien der Richtlinie des GKV-Spitzenverbandes „Begutachtungsanleitung Vorsorge und Rehabilitation“ vom 02.07.2018 definiert. Die Rekrutierung erfolgte zwischen Oktober 2020 und März 2022 in Akutkliniken der Regionen Heidelberg/Rhein-Neckar-Kreis und Bad Schönborn/Mittlerer Oberrhein.

### Datengrundlage

Grundlage der GE waren pseudonymisierte Abrechnungsdaten der AOK BW von Versicherten, die ihre Zustimmung zur Auswertung erteilten. Eine Genehmigung durch das Ministerium für Soziales, Gesundheit und Integration Baden-Württemberg zur Übermittlung von Sozialdaten nach § 75 Abs. 1 SGB X lag vor. Berücksichtigt wurden Leistungsbereiche der ambulanten und stationären Krankenhausbehandlungen, Heil- und Hilfsmittel, Reha, Arzneimittel und Pflege.

### Intervention

**Abb. 1:**
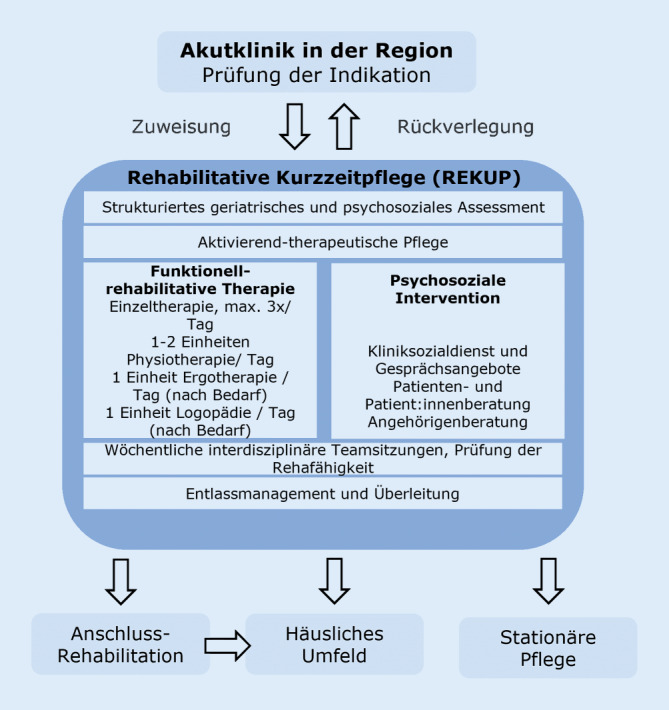
Versorgungsschema REKUP

REKUP erweitert die KZP (§ 42 SGB XI, § 39c SGB V) und umfasst neben den üblichen Versorgungsleistungen der geriatrischen Rehabilitation eine aktivierend-therapeutische Pflege mit funktionell-rehabilitativen Therapieelementen, psychosozialer Intervention sowie Beratung (Abb. 1). REKUP erfolgt trägerübergreifend und vermeidet den belastenden Wechsel zwischen Einrichtungen.

### Kontrollgruppe

Für die GE wurde eine KG aus Sekundärdaten aller Versicherten der AOK BW mittels Group-Matching (1:2) ermittelt. Einschlusskriterien waren: Versorgungsabfolge (Aufenthalt in KZP direkt im Anschluss an initialen KH-Aufenthalt), Geschlecht, ≥ 70 Jahre bei Entlassung aus REKUP/KZP und Pflegegrad (PG) 0 bis 3 vor KH-Einweisung, Diagnose-Cluster bei Entlassung aus KH-Aufenthalt. Ausschlusskriterien waren: vorliegender PG > 3, < 70 Jahre, stationäre Pflege vor initialem KH-Aufenthalt.

### Beobachtungszeitraum

**Abb. 2:**
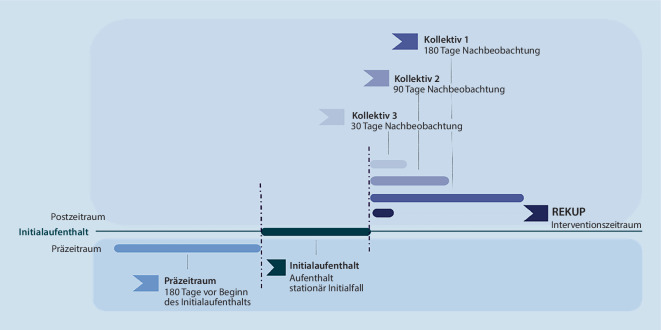
Nachbeobachtungszeitraum der Kollektive in der gesundheitsökonomische Evaluation

Für den Präzeitraum (PräZ) lagen für alle Patient:innen Daten von 180 Tagen vor. Aufgrund der Coronapandemie kam es zu Abweichungen des Vorgehens bei der Rekrutierung, sodass Patient:innen statt 180 Tage teils nur über einen verkürzten Postzeitraum (PostZ) (90 bzw. 30 Tage) nachbeobachtet wurden. So wurde die Sekundärdatenanalyse (SDA) für 3 Kollektive (K1, K2, K3) mit unterschiedlich langem Nachbeobachtungszeitraum (NBZ) durchgeführt (Abb. 2).

### Zielparameter

Primärer Outcome-Parameter für die GE auf Basis der Abrechnungsdaten war der kombinierte Endpunkt „Tage bis DP oder Versterben“ ab Entlassung aus REKUP/KZP. Zudem wurden folgende Parameter erhoben:

- Alter, Geschlecht, Todesdatum,
- Tage in REKUP (IG)/KZP (KG),
- Dauer initialer KH-Aufenthalt,
- Rehospitalisierungsrate während REKUP/KZP,
- Inanspruchnahme stationäre Rehabilitation bzw. vollstationäre DP (PostZ),
- Inanspruchnahme und Kosten je Leistungsbereich.

### (Interventions‑)Kosten

Die Kosten (in €) entsprechen den Jahren 2020–2022 gemäß vorliegender Abrechnungsdaten. Eine Diskontierung erfolgte nicht. Die durchschnittlichen Kosten wurden je Tag berechnet. Kosten für REKUP wurden auf Basis des Tagespreises der stationären geriatrischen Rehabilitation berechnet und betrugen 237,50 € pro Tag (inkl. 8 € Coronaaufschlag).

### Analyse und Auswertung

Das Vorgehen der SDA orientierte sich an der Guten-Praxis-Sekundärdatenanalyse [12]. Zur Analyse der Ressourcenverbräuche und Kosten wurden u. a. Chi^2^- bzw. Mann-Whitney-U-Tests verwendet.

Um den Effekt von REKUP auf die Gesamtkosten zu analysieren, wurde unter Verwendung des Difference-in-difference(DiD)-Ansatzes ein generalisiertes lineares Modell mit Gamma-Verteilung und Identitätslink geschätzt (Zusatzmaterial online: Supplement 1).

Inkrementelle Kosten wurden berechnet, um im Falle von statistisch signifikanten Kostenunterschieden eine KE-Analyse durchzuführen. Mittels univariater Sensitivitätsanalyse wurde der Einfluss der Höhe der Interventionskosten (± 10 %) auf das Ergebnis ermittelt.

## Ergebnisse

### Studienpopulation

In die Erprobungsstudie waren 54 Patient:innen, die im Anschluss an einen KH-Aufenthalt in REKUP gingen, eingeschlossen. Für die GE konnten 43 Patient:innen berücksichtigt werden (*n* = 6: keine Teilnahme an Evaluation; *n* = 5: fehlende Einverständniserklärung zur Datenverarbeitung).

Zudem wurden 86 Patient:innen für die KG identifiziert. Somit wurden 129 Patient:innen der AOK BW, aufgeteilt in 3 Kollektive (K1: *n* = 72 [IG1: *n* = 24 bzw. KG1: *n* = 48], K2: *n* = 108 [IG2: *n* = 36 bzw. KG2: *n* = 72], K3: *n* = 129 [IG3: *n* = 43 bzw. KG3: *n* = 86]), für die GE berücksichtigt. In K3 sind alle Patient:innen von K1 und K2 eingeschlossen, jeweils mit entsprechend verkürztem NBZ bzw. in K2 alle Patient:innen des K1.

Studiencharakteristika je Kollektiv sind in Tab. 1 dargestellt. Unterschiede wiesen IG und KG in allen Kollektiven bei der Zahl der im PostZ Verstorbenen auf (*p* < 0,05) sowie der Dauer des initialen KH-Aufenthalts (K1/K3: *p* < 0,05; K2: *p* < 0,01), wobei die IG jeweils eine längere Aufenthaltsdauer hatte.

**Tab. 1:** Charakteristika jeKollektiv

	IG1	KG1	IG2	KG2	IG3	KG3
*n*	24	48	36	72	43	86
*Alter zum Zeitpunkt der Aufnahme in REKUP/KZP* MW (±) in Jahren	80,7 (6,0)	81,4 (5,8)	81,3 (6,1)	82,2 (5,7)	82,2 (6,0)	82,7 (5,7)
*Weiblich* *n* (%)	16 (66,7)	32 (66,7)	25 (69,4)	50 (69,4)	30 (69,8)	60 (69,8)
*Aufenthaltsdauer in der REKUP/KZP*^1^ MW in Tagen (Min.; Max)	20,5 (11; 22)	20,4 (0; 86)	20,8 (11; 30)	22,8 (0; 86]	19,6 (2; 30)	18,7 (0; 30)
*Dauer initialer KH-Aufenthalt*^1^ MW in Tagen (Min.; Max.)	22,8* (5; 74)	20,4* (1; 96)	24,1^Ɨ^ (5; 103)	15,3^Ɨ^ (1; 96)	22,5* (5; 103)	14,7* (1; 96)
*Patient:innen mit Rehospitalisierung(en) während der REKUP/KZP* *n* (%)	4 (16,6)	17 (35,4)	7 (19,4)	24 (33,3)	10 (23,3)	27 (31,4)
*Patient:innen mit Rehaaufenthalt (PostZ)*^1^ *n* (%)	20^ǂ^ (83,3)	7^ǂ^ (14,6)	32^ǂ^ (88,9)	11^ǂ^ (15,3)	34^ǂ^ (79,1)	7^ǂ^ (8,1)
*Verstorbene (PostZ)*^1^ *n* (%)	3* (12,5)	13* (27,1)	1* (2,8)	15* (20,8)	1* (2,3)	12* (14,0)
*Tage im Nachbeobachtungszeitraum* MW (Min.; Max.)	171,6 (40; 180)	145,2 (2; 180)	88,6 (40; 90)	79,5 (2; 90)	29,7 (19; 30)	28,1 (2; 30)
*Patient:innen in vollstationärer DP (PostZ)*^1^ *n* (%)	1^ǂ^ (4,2)	26^ǂ^ (54,2)	4^ǂ^ (11,1)	43^ǂ^ (59,7)	1^ǂ^ (2,3)	29^ǂ^ (33,7)
*Tage bis DP/Versterben*^1^ MW (Min.; Max.)	167,6^ǂ^ (40; 180)	82,0^ǂ^ (2; 180)	85,2^ǂ^ (20; 90)	48,9^ǂ^ (2; 90)	29,5^ǂ^ (19; 30)	25,4^ǂ^ (2; 30)

*DP* Dauerpflege, *IG* Interventionsgruppe, *KG* Kontrollgruppe, *KH* Krankenhaus, *KZP* Kurzzeitpflege, *MW* Mittelwert, *PostZ* Postzeitraum, *REKUP* Rehabilitative Kurzzeitpflege, *±* Standardabweichung

^1^Überprüfung auf statistisch signifikante Unterschiede mittels Mann-Whitney-U-Test: *p < 0,05; ^Ɨ^*p* < 0,01; ^ǂ^*p* < 0,001

### Klinische Outcomes

Die durchschnittliche Anzahl an Tagen bis zu einer stationären DP oder Versterben war in allen Kollektiven statistisch signifikant unterschiedlich (*p* < 0,001) zugunsten der jeweiligen IG. Auch bei Patient:innen mit Rehaaufenthalt und stationärer DP zeigten sich in allen Kollektiven statistisch signifikante Unterschiede (*p* < 0,001) zugunsten der IG.

### Kosten

Tab. 2 zeigt die durchschnittlichen Gesamtkosten für jedes Kollektiv je Zeitraum. Die durchschnittlichen Kosten je Leistungsbereich finden sich im Zusatzmaterial online: Supplement 2.

**Tab. 2:** Durchschnittliche Gesamtkosten

Kosten je Tag in €	IG1 (*n* = 24)		KG1 (*n* = 48)		IG2 (*n* = 36)		KG2 (*n* = 72)		IG3 (*n* = 43)		KG3 (*n* = 86)	
	MW (±)	Min; Max	MW (±)	Min; Max	MW (±)	Min; Max	MW (±)	Min; Max	MW (±)	Min; Max	MW (±)	Min; Max
*Gesamtkosten, PräZ*	373,7* (629,5)	1,1; 2529	188,8* (521,3)	0,02; 3473	395,7 (702,4)	2; 2838	235,6 (505,0)	6; 3482	403,2 (673,4)	1; 2838	223,7 (481)	3; 3477
*Initialer KH-Aufenthalt*	733,7 (440,6)	338; 1803	592,9 (304,3)	207; 1748	697,2 (481,2)	481; 305	595,8 (331,0)	178; 1748	644,8 (380,8)	305; 1803	586,8 (322,8)	178; 1748
*Gesamtkosten, PostZ*	518,5 (725,6)	55; 3475	538,2 (852,2)	37; 5532	440,7 (517,1)	108; 2958	395,2 (682,8)	46; 5532	331,8 (282,8)	25; 2034	375,2 (616,1)	70; 5532
*(Interventions‑)Kosten REKUP/KZP*	4858,9* (542)	2613; 5225	1727,2* (986)	204; 5103	4928,2* (596,5)	2613; 7125	1888,9* (1089)	64; 5444	4650,6* (1185,6)	475; 7125	1581,3* (650,4)	64; 3277

*IG* Interventionsgruppe, *KG* Kontrollgruppe, *KZP* Kurzzeitpflege, *MW* Mittelwert, *PostZ* Postzeitraum, *PräZ* Präzeitraum, *REKUP* rehabilitative Kurzzeitpflege, *±* Standardabweichung

Überprüfung auf statistisch signifikante Unterschiede mittels Mann-Whitney-U-Test: *p < 0,05

Die durchschnittlichen Gesamtkosten je Tag der IG1 waren im PräZ doppelt so hoch wie die der KG1 (IG1: 374 €; KG1: 189 €; *p* < 0,05). In K2 und K3 zeigten sich im PräZ keine statistisch signifikanten Unterschiede der Gesamtkosten zwischen IG und KG. Auf Ebene der einzelnen Leistungsbereiche waren lediglich die Arzneimittelkosten je Tag der KG2 im PräZ statistisch signifikant höher als die Kosten der IG2 (IG2: 4 €; KG2: 10 €; *p* < 0,01). Die durchschnittlichen Kosten je Tag des initialen KH-Aufenthalts waren in der IG höher als in der KG ohne statistisch signifikante Unterschiede. Für die durchschnittlichen Gesamtkosten je Tag im PostZ zeigten sich in keinem Kollektiv statistisch signifikante Unterschiede; sie lagen über alle Gruppen zwischen 332 € (IG3) und 538 € (KG1).

Für Pflegeleistungen (ohne Kosten des initialen KZP-Aufenthalts) entstanden im PostZ für alle Kollektive statistisch signifikant höhere durchschnittliche Kosten je Tag der KG im Vergleich zur IG (beispielsweise K1: IG1: 18 €; KG1: 35 €; *p* < 0,001), ebenso wie für Arzneimittel (beispielsweise K1: IG1: 5 €; KG1: 17 €; *p* < 0,001). Zudem waren die Kosten je Tag für Heil‑/Hilfsmittel der KG im K3 statistisch signifikant höher (IG3: 69 €; KG3: 178 €; *p* < 0,001).

Im PostZ entstanden in allen Kollektiven statistisch signifikant höhere durchschnittliche Kosten pro Tag in der IG für stationäre Reha (beispielsweise K3: IG3: 62 €; KG3: 10 €; *p* < 0,001), wie auch höhere Interventionskosten für REKUP im Vergleich zu den Kosten des KZP-Aufenthalts der KG (K1: ∆ 3132 €; K2: ∆ 3039 €; K3: ∆ 3069 €; *p* < 0,05).

Die DiD-Analyse zeigte, dass die durchschnittlichen Gesamtkosten pro Tag in der IG im Vergleich zur KG niedriger sind (K1: ∆ −205 €; K2: ∆ −115 €; K3: ∆ −223 €; Zusatzmaterial online: Supplement 1). Die Zugehörigkeit zur IG hatte einen statistisch signifikanten Einfluss auf die Höhe der Gesamtkosten je Tag im K3 (*p* < 0,05).

### Sensitivitätsanalyse

Bei einer 10 %igen Erhöhung bzw. Verringerung der Interventionskosten ergab die DiD-Analyse, dass im K3 die Zugehörigkeit zur IG einen statistisch signifikanten Einfluss auf die Gesamtkosten pro Tag bei Senkung der Kosten von REKUP um 10 % hat (*p* < 0,05).

## Diskussion

Die Analyse der Kosten von GKV-Versicherten mit REKUP bzw. KZP nach initialem KH-Aufenthalt weist auf einen Vorteil der neuen Versorgungsform hinsichtlich poststationärer Inanspruchnahme von Rehabilitation und stationärer DP hin. Auch konnte eine Vorteilhaftigkeit anhand des Endpunkts „Tage bis DP oder Versterben“ gezeigt werden. Die deskriptive Analyse der Gesamtkosten je Tag im PostZ ergab in keinem Kollektiv statistisch signifikante Unterschiede zwischen IG und KG, trotz statistisch signifikant höherer Interventionskosten. In der DID-Analyse zeigte sich für das K3, dass die Zugehörigkeit zur IG einen statistisch signifikanten Einfluss auf die Höhe der durchschnittlichen Gesamtkosten je Tag hatte. Dabei ist zu berücksichtigen, dass im K3 der NBZ 30 Tage ab Entlassung aus initialem KH-Aufenthalt betrug. Vor dem Hintergrund, dass geriatrische Patient:innen im Anschluss durchschnittlich 20 Tage institutionalisiert waren, ist die Aussagekraft dieses Ergebnisses mit Einschränkungen verbunden. Die Sensitivitätsanalyse ergab in den Kollektiven 1 und 2 keinen statistisch signifikanten Interventionseffekt auf die Gesamtkosten pro Tag. Für das K3 zeigte sich ein statistisch signifikanter Interventionseffekt auf die Gesamtkosten pro Tag, wenn die Interventionskosten um 10 % gesenkt wurden (*p* < 0,05). Bei statistisch signifikanten Vorteilen des klinischen Outcome-Parameters und gleichzeitig geringeren oder gleichen Kosten entfällt die Berechnung einer inkrementellen Kosteneffektivitätsrelation im Sinne einer Kostenminimierungsanalyse.

REKUP ist auf den deutschen Versorgungskontext ausgerichtet und hat das Ziel, im Anschluss an einen KH-Aufenthalt Rehabilitationsfähigkeit zu erreichen und eine Entlassung ins häusliche Umfeld zu ermöglichen. International vergleichbare Ansätze sind „Transitional-care“-Modelle (TCM), für die gezeigt werden konnte, dass Kosteneinsparungen möglich sind [7], wobei eine in Regensburg erprobte, an das TCM angelehnte Intervention keinen statistisch signifikanten Effekt auf die Kosten zeigte [4]. Aufgrund von regionalen Besonderheiten der Geriatriekonzepte und der damit verbundenen Organisation der Rehabilitation sowie möglichen bestehenden Fehlanreizen im Finanzierungssystem der geriatrischen Rehabilitation [9] ist die Klärung einer Finanzierung und Vergütung einer solchen Maßnahme essenziell. Die vorliegende auf Routinedaten basierenden GE konnte zeigen, dass es Hinweise auf Vorteile der REKUP hinsichtlich der Inanspruchnahme von Rehabilitation, der Vermeidung von stationärer DP und Versterben bei gleichen Kosten gibt.

### Limitationen

Die Erprobung von REKUP während der Coronapandemie führte zu einer geringeren Einschlussrate und verkürzten NBZ, wodurch nicht alle anschließenden Kosten berücksichtigt wurden. Auch lagen keine ambulant ärztlichen Abrechnungsdaten vor, und etwaige Kostenentwicklungen in diesem Bereich konnten nicht analysiert werden.

Hinzu kommt, dass im PräZ die KG1 weniger Leistungen in Anspruch nahm und statistisch signifikant geringere Kosten als IG1 aufwies. Im PostZ zeigten sich statistisch signifikant höhere Kosten der KG in den Bereichen Pflege und Arzneimittel, und mehr Patient:innen gingen in DP oder verstarben. Insbesondere hinsichtlich des Kostenunterschiedes im Bereich der Arzneimittel konnte eine Verbindung zur Morbidität aufgrund der fehlenden ambulant ärztlichen Abrechnungsdaten nicht hergestellt werden. Insgesamt lassen diese Aspekte jedoch auf eine potenziell höhere Morbidität der KG im PostZ bei gleichzeitig geringerer Morbidität im PräZ schließen. Mögliche Erklärungen für diese Heterogenität zwischen IG und KG sind die angewandten Kriterien beim Matching. So konnte ein Rehabilitationsbedarf zum Zeitpunkt der Entlassung bei gleichzeitig noch nicht vorhandener Rehabilitationsfähigkeit nicht berücksichtigt werden, da ein vorhandenes Rehabilitationspotenzial anhand der Abrechnungsdaten nicht ermittelt hätte werden können. Somit ist möglich, dass die KG trotz ähnlicher Hauptdiagnose des initialen KH-Aufenthalts in einem schlechteren Zustand entlassen wurden. Beim Matching auf zweistelliger Ebene der ICD-Klassifikation wurde auch nicht für mögliche Begleiterkrankungen wie Demenz und den kognitiven Status, welche Ausschlusskriterien für die IG waren, kontrolliert. Auch die abgerechnete DRG war kein Kriterium des Matching, was die Vergleichbarkeit hinsichtlich Dauer und Kosten des initialen stationären KH-Aufenthalts verbessern hätte können. Aufgrund dieser Einschränkungen könnte der Effekt von REKUP tendenziell überschätzt werden.

## Fazit für die Praxis

- Der zu erwartende Anstieg Pflegebedürftiger erfordert Versorgungsformen, die funktionelle Einschränkungen stabilisieren und Pflegebedürftigkeit vermeiden bzw. verringern.
- Die Evaluation der Erprobung von REKUP auf Basis von Routinedaten zeigt Hinweise auf Vorteile gegenüber der KZP hinsichtlich der Überleitung in Rehabilitation und der Vermeidung von DP bei gleichen durchschnittlichen Kosten.
- Aufgrund methodischer Einschränkungen ist weitere Forschung erforderlich, um den Einfluss auf Kosten und klinische Ergebnisse zu untersuchen.

## Supplementary Information

Supplement 1: Durchschnittliche Kosten je Kollektiv je Leistungsbereich

Supplement 2: Ergebnisse der generalisierten linearen Modelle
