# Supplementary material for: Health economic evaluation of rehabilitative short-term care: Analysis of the costs of geriatric patients with and without rehabilitative short-term care after inpatient hospital stay
Source: Z Gerontol Geriatr. 2024 May 15;57(6):475–81. [Article in German] doi: 10.1007/s00391-024-02307-2 (PMC11422432; doi:10.1007/s00391-024-02307-2)
Supplement: Supplementary file 1 — Supplement 1: Durchschnittliche Kosten je Kollektiv je Leistungsbereich [file 391_2024_2307_MOESM1_ESM.docx]

## Supplement

Supplement 1: Durchschnittliche Kosten je Kollektiv je Leistungsbereich

| **Kosten je Tag in €** | **IG1** | | | **KG1** | | | **IG2** | | | **KG2** | | | **IG3** | | | **KG3** | | |
| --- | --- | --- | --- | --- | --- | --- | --- | --- | --- | --- | --- | --- | --- | --- | --- | --- | --- | --- |
|  | **n^1^**  **(%)** | **MW**  **(** **± )** | **Min; Max** | **n^1^**  **(%)** | **MW**  **(** **± )** | **Min; Max** | **n^1^**  **(%)** | **MW**  **(** **± )** | **Min; Max** | **n^1^**  **(%)** | **MW**  **(** **± )** | **Min;**  **Max** | **n^1^**  **(%)** | **MW**  **(** **± )** | **Min; Max** | **n^1^ (%)** | **MW**  **(** **± )** | **Min; Max** |
| **Prä-Zeitraum** | | | | | | | | | | | | | | | | | | |
| **Gesamt-kosten** | 24  (100) | 373,7*  (629,5) | 1,1; 2.529 | 48  (100) | 188,8*  (521,3) | 0,02; 3.473 | 36  (100) | 395,7  (702,4) | 2; 2.838 | 72  (100) | 235,6  (505,0) | 6;  3.482 | 43  (100) | 403,2  (673,4) | 1; 2.838 | 86  (100) | 223,7  (481) | 3; 3.477 |
| **Krankenhaus** | 14  (58) | 43,0  (63,3) | 0;  232 | 20  (42) | 22,7  (39,9) | 0;  172 | 29  (81) | 34,9  (54,9) | 0;  232 | 38  (53) | 22,7  (38,9) | 0;  179 | 22  (51) | 37,1  (56,6) | 0; 232 | 39  (45) | 20,6  (36,9) | 0; 179 |
| **Stat. Reha** | 2  (8) | 1,0  (4,7) | 0; 23 | 2  (4) | 0,7  (4,4) | 0;  31 | 3  (8) | 1,1  (4,4) | 0;  23 | 5  (7) | 1,9  (9,2) | 0;  67 | 5  (12) | 2,1  (0,0) | 0; 27 | 6  (7) | 1,7  (8,4) | 0; 67 |
| **Heil- /Hilfs-mittel** | 22  (92) | 316,7  (610,5) | 0;  2.467 | 47  (98) | 181,2  (518,4) | 0;  3.455 | 34  (94) | 344,5  (690,6) | 0; 2.821 | 70  (97) | 192,0  (497,6) | 0;  3.455 | 39  (91) | 348,4  (664,3) | 0; 2.821 | 84  (98) | 186,3  (473,1) | 0; 3.455 |
| **Arzneimittel** | 23  (96) | 5,0  (4,7) | 0;  18 | 46  (96) | 5,1  (5,6) | 0; 28 | 35  (97) | 4,3**^ǂ^**  (4,0) | 0;  18 | 72  (100) | 10,0**^ǂ^**  (9,5) | 0,30; 68 | 42  (98) | 4,3  (3,9) | 0; 18 | 84  (98) | 5,2  (6,7) | 0; 49 |
| **Amb. KH** | 2  (8) | 0,1  (0,3) | 0; 1 | 8  (17) | 0,6  (2,6) | 0; 17 | 4  (11) | 0,1  (0,3) | 0;  1 | 12  (17) | 0,5  (2,2) | 0;  17 | 8  (19) | 0,2  (0,5) | 0; 3 | 12  (14) | 0,4  (2,0) | 0; 17 |
| **Pflege** | 14  (58) | 7,9  (8,8) | 0; 28 | 31  (65) | 6,8  (6,9) | 0; 19 | 23  (64) | 10,8  (14,1) | 0;  68 | 50  (69) | 8,5  (14,2) | 0;  113 | 28  (65) | 11,1  (13,9) | 0; 68 | 60  (70) | 9,5  (14,0) | 0; 113 |
| **Initialaufenthalt** | | | | | | | | | | | | | | | | | | |
| **Krankenhaus** | 24  (100) | 733,7  (440,6) | 338; 1.803 | 48  (100) | 592,9  (304,3) | 207; 1.748 | 36  (100) | 697,2  (481,2) | 481; 305 | 72  (100) | 595,8  (331,0) | 178;  1.748 | 43  (100) | 644,8  (380,8) | 305; 1.803 | 86  (100) | 586,8  (322,8) | 178; 1.748 |
| **Post-Zeitraum** | | | | | | | | | | | | | | | | | | |
| **Gesamt-kosten** | 24  (100) | 518,5  (725,6) | 55;  3.475 | 48  (100) | 538,2  (852,2) | 37; 5.532 | 36  (100) | 440,7  (517,1) | 108; 2.958 | 72  (100) | 395,2  (682,8) | 46;  5.532 | 43  (100) | 331,8  (282,8) | 25; 2.034 | 86  (100) | 375,2  (616,1) | 70; 5.532 |
| **Krankenhaus** | 11  (46) | 32,9  (46,0) | 0;  125 | 32  (67) | 94,9  (169,4) | 0; 788 | 14  (39) | 33,4  (49,6) | 0;  159 | 40  (56) | 87,7  (149,1) | 0;  788 | 8  (19) | 36,1  (82,4) | 0; 298 | 35  (41) | 91,9  (159,1) | 0; 788 |
| **Stat. Reha** | 20  (83) | 27,5**^ǂ^**  (23,2) | 0;  114 | 7  (15) | 5,2**^ǂ^**  (14,0) | 0; 61 | 32  (89) | 51,8**^ǂ^**  (28,3) | 0; 124 | 11  (15) | 9,9**^ǂ^**  (24,8) | 0;  107 | 34  (79) | 61,6**^ǂ^**  (44,7) | 0; 230 | 7  (8%) | 10,1**^ǂ^**  (35,2) | 0; 168 |
| **Heil- /Hilfs-mittel** | 24  (100) | 404,9  (731,9) | 6;  3.394 | 48  (100) | 364,4  (727,0) | 2; 4.534 | 35  (97) | 278,6  (519,5) | 0; 2.797 | 72  (100) | 224,2  (563,5) | 0,5; 4.534 | 7  (16) | 68,5**^ǂ^**  (267,2) | 0; 1.732 | 86  (100) | 177,5**^ǂ^**  (504,6) | 0,5; 4.534 |
| **Arzneimittel** | 23  (96) | 4,7Ɨ  (3,9) | 0; 12 | 47  (98) | 17,1Ɨ  (39,2) | 0; 237 | 34  (94) | 4,0**^ǂ^**  (3,6) | 0; 15 | 70  (97) | 12,5**^ǂ^**  (18,5) | 0;  114 | 7  (16) | 1,4**^ǂ^** (5,0) | 0; 28 | 83  (97) | 18,0**^ǂ^**  (29,5) | 0; 182 |
| **Amb. KH** | 3  (13) | 0,2  (0,5) | 0; 2 | 3  (6) | 0,3  (1,4) | 0; 10 | 2  (6) | 0,1  (0,4) | 0; 2 | 7  (10) | 0,3  (1,3) | 0; 10 | 0  (0,0) | - | - | 4  (5) | 0,3  (1,3) | 0; 10 |
| **Pflege** | 18  (75) | 18,3**^ǂ^**  (18,0) | 0; 55 | 47  (98) | 35,2**^ǂ^** (50,2) | 0; 335 | 29  (81) | 16,2**^ǂ^**  (15,3) | 0; 56 | 72  (100) | 31,5**^ǂ^**  (41,6) | 0; 335 | 27  (63) | 8,9**^ǂ^**  (9,7) | 0; 47,9 | 80  (93) | 19,8**^ǂ^**  (36,9) | 0; 335 |
| **Interventionskosten REKUP (IG) / Kosten KZP (KG) [**alle Versicherten**]** | | | | | | | | | | | | | | | | | | |
| **Kosten REKUP bzw. KZP** | 24  (100) | 4.858,9*  (542) | 2.613; 5.225 | 48  (100) | 1.727,2*  (986) | 204; 5.103 | 36  (100) | 4.928,2*  (596,5) | 2.613; 7.125 | 72  (100) | 1.888,9*  (1.089) | 64;  5.444 | 43  (100) | 4.650,6*  (1.185,6) | 475; 7.125 | 86  (100) | 1.581,3*  (650,4) | 64; 3.277 |

***^1^*** *Anzahl Versicherte mit mind. 1 Leistung*

IG: Interventionsgruppe; KG: Kontrollgruppe; KZP: Kurzzeitpflege; MW: Mittelwert; REKUP: rehabilitative Kurzzeitpflege; SD: Standardabweichung.

Überprüfung auf statistisch signifikante Gruppenunterschiede mittels Mann-Whitney-U-Test: *: p<0,05; Ɨ: p<0,01; ǂ: p<0,001);
