# Supplementary material for: Health economic evaluation of rehabilitative short-term care: Analysis of the costs of geriatric patients with and without rehabilitative short-term care after inpatient hospital stay
Source: Z Gerontol Geriatr. 2024 May 15;57(6):475–81. [Article in German] doi: 10.1007/s00391-024-02307-2 (PMC11422432; doi:10.1007/s00391-024-02307-2)
Supplement: Supplementary file 2 — Supplement 2: Ergebnisse der generalisierten linearen Modelle [file 391_2024_2307_MOESM2_ESM.docx]

## Supplement

Supplement 2: Ergebnisse der generalisierten linearen Modelle

|  | **Koef.** | **Std.Fehler** | **p-Wert** | **95%-Konfidenzintervall** | | |
| --- | --- | --- | --- | --- | --- | --- |
| **K1** | | | | | | |
| Zeit [PostZ] | 349,42 | 107,67 | 0,001 | 118,91 | | 258,60 |
| Gruppe [IG] | 184,90 | 105,94 | 0,081 | -22,74 | | 392,53 |
| Gruppe * Zeit [IG * PostZ] | -204,60 | 201,76 | 0,311 | -600,05 | | 190,85 |
| Konstante | 188,76 | 35,64 | <0,001 | 118,91 | | 258,60 |
| **K2** | | | | | | |
| Zeit [PostZ] | 159,62 | 63,48 | 0,012 | | 35,21 | 284,03 |
| Gruppe [IG] | 160,11 | 83,77 | 0,056 | | -4,06 | 324,30 |
| Gruppe * Zeit [IG * PostZ] | -114,62 | 131,84 | 0,385 | | -373,04 | 143,78 |
| Konstante | 235,60 | 32,50 | <0,001 | | 171,89 | 299,30 |
| **K3** | | | | | | |
| Zeit [PostZ] | 151,49 | 53,45 | 0,005 | 46,73 | | 256,25 |
| Gruppe [IG] | 179,55 | 74,96 | 0,017 | 32,64 | | 326,46 |
| Gruppe * Zeit [IG * PostZ] | -222,91 | 104,99 | 0,034 | -428,69 | | -17,13 |
| Konstante | 223,69 | 27,37 | <0,001 | 170,04 | | 277,33 |

IG: Interventionsgruppe; PostZ: Post-Zeitraum. Std.Fehler: Standardfehler.

Methodische Erläuterung: Abhängige Variable Y waren die Gesamtkosten pro Tag und als Kovariate wurden die Dummy-Variablen „Gruppenzugehörigkeit“ (IG/KG) und „Zeitraum“ (PräZ/PostZ) sowie der Interaktionsterm dieser beiden Variablen (Gruppe*Zeit) aufgenommen. Der Schätzer β3 des Interaktionsterms ist der DiD-Schätzer und repräsentiert den Interventionseffekt auf die Kosten:
$Y_{it}=\beta_{0}+\beta_{1}*{Gruppe}_{i}+\beta_{2}*{Zeit}_{it}+\beta_{3}*\left( {Gruppe}_{i}*{Zeit}_{it} \right)+\varepsilon_{it}$
